# Approaching a universal scaling relationship between fracture stiffness and fluid flow

**DOI:** 10.1038/ncomms10663

**Published:** 2016-02-12

**Authors:** Laura J. Pyrak-Nolte, David D. Nolte

**Affiliations:** 1 Department of Physics and Astronomy, Purdue University, 525 Northwestern Avenue, West Lafayette, Indiana 47907-2036, USA; 2 Lyle School of Civil Engineering, Purdue University, West Lafayette, Indiana 47907-0395, USA; 3 Department of Earth, Atmospheric and Planetary Sciences, Purdue University, West Lafayette, Indiana 47907-2051, USA

## Abstract

A goal of subsurface geophysical monitoring is the detection and characterization of fracture alterations that affect the hydraulic integrity of a site. Achievement of this goal requires a link between the mechanical and hydraulic properties of a fracture. Here we present a scaling relationship between fluid flow and fracture-specific stiffness that approaches universality. Fracture-specific stiffness is a mechanical property dependent on fracture geometry that can be monitored remotely using seismic techniques. A Monte Carlo numerical approach demonstrates that a scaling relationship exists between flow and stiffness for fractures with strongly correlated aperture distributions, and continues to hold for fractures deformed by applied stress and by chemical erosion as well. This new scaling relationship provides a foundation for simulating changes in fracture behaviour as a function of stress or depth in the Earth and will aid risk assessment of the hydraulic integrity of subsurface sites.

## Abstract (web summary)

Fractures in rock can be altered geochemically and deformed under stress, affecting fluid flow rates across many orders of magnitude. Here, the authors present a universal scaling relationship between fluid flow and fracture specific stiffness, which will aid the interpretation of subsurface sites.

## Graphical abstract

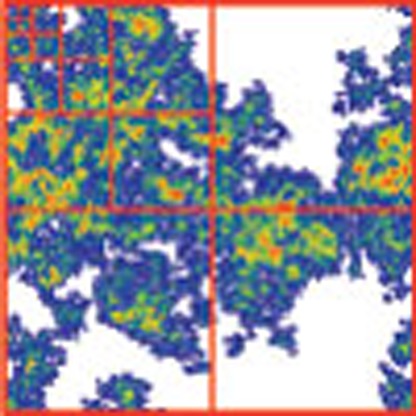

Fractures in rock provide the dominant conductive pathways for fluids to move through the Earth's crust, influencing a wide range of subsurface human activities including the extraction of hydrocarbons, the sequestration of green house gases and the protection of aquifers. However, fractures are intrinsically heterogeneous and easily modified by natural and human processes. They may be altered geochemically and deformed under stress, affecting fluid flow rates that can vary across many orders of magnitude.

For hydrologic purposes, a fracture may be viewed as a quasi-two-dimensional (2D) network of void spaces through which fluids flow. Volumetric flow rates are controlled by the size and spatial distribution of the apertures of the void space^1^,^2^,^3^. On the other hand, the mechanical properties of a fracture are controlled by the asperities, which are the discrete points of contact between the two fracture surfaces. Apertures and asperities are complementary aspects of the same fracture geometry and each represents an influence network. The connected apertures define a percolation network, whereas the discrete points of contact are connected through the rock matrix as a separate stress network that controls the mechanical deformation of a fracture. These two networks combine to provide a full description of the fracture geometry, which is the nexus between the hydraulic and mechanical properties of a fracture. However, as demonstrated by many investigations, fracture geometry is complex and sensitive to alterations^4^,^5^,^6^,^7^.

Recently, Petrovitch *et al.*^8^ demonstrated numerically that fracture-specific stiffness is an effective parameter that captures the deformed topology of a fracture and can be used as the basis of a scaling relationship for fluid flow through fractures. This was an important development, because fracture-specific stiffness can be estimated from seismic wave attenuation and velocity^9^,^10^,^11^,^12^,^13^,^14^. The Petrovitch relationship holds for random distributions of weakly correlated apertures, but it remained an open question whether this relationship would be universal, that is, whether it would apply to spatially correlated aperture distributions or to fractures with preferential erosion that causes channelized flow.

In this study we demonstrate that a scaling relationship exists that accounts for spatial correlations in fracture aperture distributions and also captures the behaviour of channelized flow within a fracture. This relationship can be used as a guide for incorporating the appropriate behaviour of fractures into continuum models of the subsurface where stresses vary with depth. This relationship also provides a path forward to the ultimate goal of remotely monitoring fluid flow or relative fluid flow among fractures in the Earth's subsurface.

## Results

### Fracture void topology

**Figure 1:**
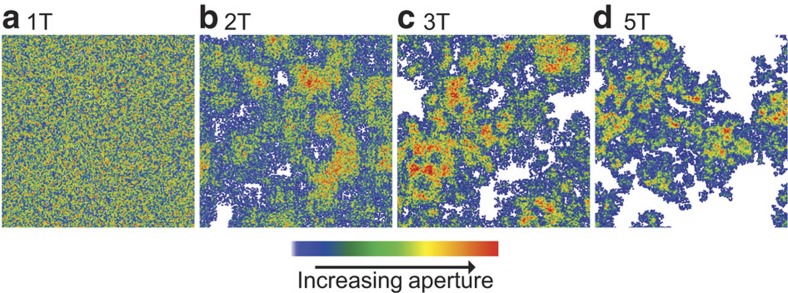
Simulated fracture aperture distributions. Aperture distributions are shown for (**a**) an uncorrelated aperture distribution (1T) and, with increasing correlation strength, (**b**) 2T, (**c**) 3T and (**d**) 5T. White represents contact area and increasing aperture is represented by blue through red.

For a relationship between fracture properties to be considered ‘universal' and ‘scalable', a wide variety and a large number of fracture aperture distributions must be analysed as a function of observation scale. A stratified percolation approach was used to generate spatially correlated pore-scale (micrometres) fracture void geometries for fractures that spanned in length from 0.0625 to 1.0 m (refs 15, 16). Ensembles of fractures with differing spatial correlations but with similar void volumes were generated and are referred to as 1T, 2T, 3T and 5T (Fig. 1a–d). Details of the generation are given in the Methods section. Correlations in these fractures are homogeneous and isotropic by construction (see Supplementary Fig. 1 and Supplementary Table 1).

**Figure 2:**
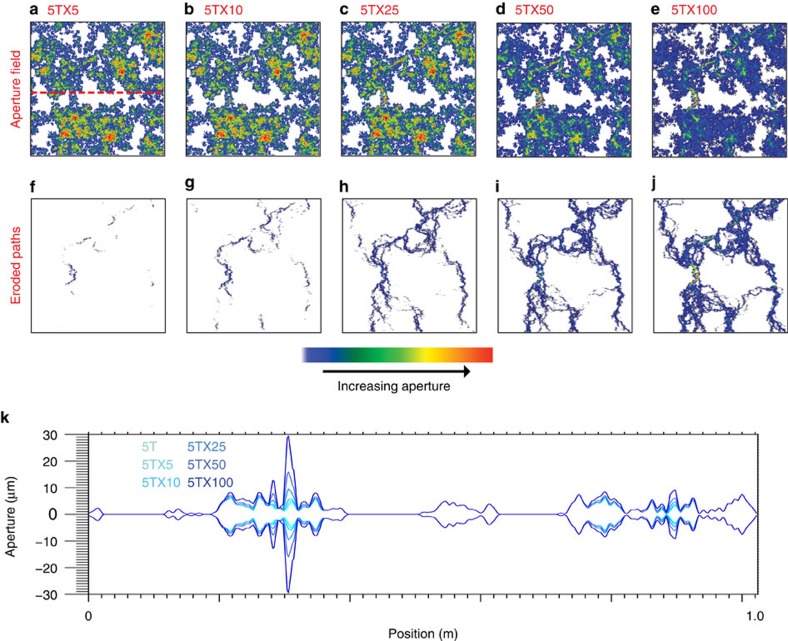
Simulated aperture distribution of chemically eroded fractures. (**a**–**e**) The aperture fields are shown for preferentially eroded fractures, (**f**–**j**) the eroded flow paths and (**k**) cross-sections from aperture fields showing the local effect of increasing chemical erosion.

To test whether systematic alterations of fracture apertures can retain the same universal scaling, the 5T aperture fields were subjected to simulated chemical erosion. Chemical erosion of a fracture can exhibit a range of behaviour depending on the relative time scales associated with advection and chemical reactions^17^,^18^,^19^. In the numerical erosion process, the amount of erosion was made proportional to the fluid flux through a given aperture^17^. This approach erodes the critical path (the path of highest aperture) and strongly affects the critical neck (the smallest aperture along this path) (see Supplementary Fig. 2 and Supplementary Tables 2 and 3). Although the fracture void volume increased by 0.3–27% with increasing erosion factors (5 × –100 ×, respectively), the critical neck/aperture increased by 55–163% because of the erosion. Examples of eroded aperture distributions are shown in Fig. 2a–e along with the eroded path (Fig. 2f–j) and a cross-section that shows the effect of preferential erosion on the apertures (Fig. 2k). As erosion increases, the aperture distribution evolves into channelized paths of large aperture.

For each fracture type shown in Figs 1 and 2, 100 patterns were generated at the 1-m scale. Each pattern was then sectioned into fracture lengths of 0.5, 0.25, 0.125 and 0.0625, m from which 200, 400, 800 and 1,600 sub-patterns, respectively, were studied (see Supplementary Fig 3). For each scale and all realizations, fracture deformation as a function of stress was simulated using a combined conjugate-gradient method and fast-multipole method^8^,^20^,^21^. A flow network model was used on the deformed fractures, to simulate fluid flow, fluid velocity and fluid pressures within a fracture^22^,^23^,^24^ as a function of stress (see Methods and Supplementary Methods for details).

### Scale dependence of flow and fracture-specific stiffness

**Figure 3:**
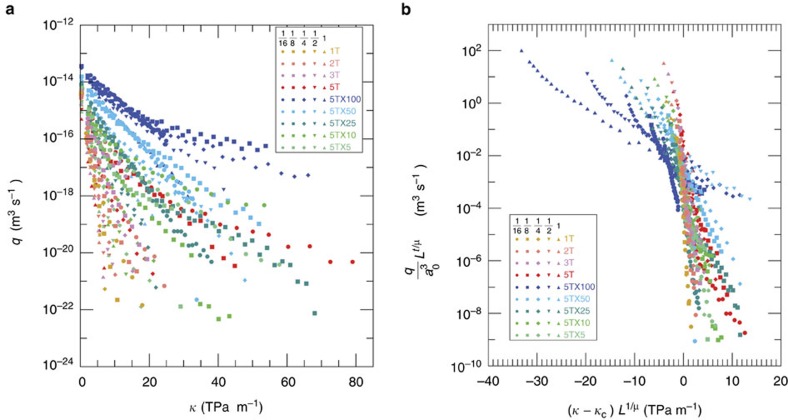
Flow and fracture-specific stiffness as a function of scale. (**a**) Flow as a function of fracture stiffness for spatially correlated patterns and eroded patterns for fracture length scales of 0.0625–1 m. (**b**) Scaled flow-stiffness data using equation (1), the Petrovitch relationship. The shape of the symbol indicates the fracture length scale: 0.0625 (circles), 0.125 (squares), 0.25 (diamonds), 0.5 (filled inverted triangle) and 1 m (filled triangle).

Average values of fluid flow, fracture displacement and void area as functions of stress were calculated for a series of scales for each pattern used in the study. Strong scale dependence is observed in the unscaled flow versus fracture-specific stiffness relationship (Fig. 3a) with small-scale fractures supporting more flow than large-scale fractures. There is a strong dependence of flow on spatial correlations as well. Fracture realizations 1T, 2T, 3T and 5T were generated with similar void volumes but with different spatial distributions that strongly affect the mean apertures, the critical necks and the contact areas. The average mean aperture of the 2T, 3T and 5T models for 1-m scale fractures were larger by 5%, 22% and 36%, respectively, relative to the 1T fracture realizations, while the average critical neck changed by +9.5%, +21% and −28% for those same patterns. The smaller critical neck for the strongly spatially correlated fractures (5T) relative to the weakly correlated fractures (1T) occurs because the increase in contact area results in a void topology similar to isthmuses, as narrow strips of land (narrow voids in this case) connect larger land areas (large areas of voids), and narrow regions of voids tend to have smaller apertures.

Strong preferential erosion (Fig. 2c–e) results in radical deviations in behaviour compared with that for the uneroded fractures (Fig. 1d) and is marked by five characteristics as follows: anisotropic aperture distributions provide enhanced connectivity between the inlet and outlet of the fracture; enhanced flow rates occur because of the larger apertures along the dominant path and the larger aperture of the critical neck (Fig. 3a); the larger void volume results in more compliant fractures even though the contact area remains the same; channelized flow paths require very high stresses to close off flow along the critical path; and a reduction of scale dependence (Fig. 3a) is observed for the correlated fractures when the flow becomes channelized and flow becomes quasi-one dimensional.

## Discussion

The importance of fracture topology is evident in the unscaled flow–stiffness relationship (Fig. 3a) and demonstrates that void volume, aperture distributions, contact area and fracture length are all essential to the hydraulic and mechanical responses of a fracture. On the other hand, it is the fracture void geometry that intimately links the mechanical and hydraulic response of a fracture and provides a means to capture the complexity of fluid flow and stiffness in a single universal relationship. Petrovitch *et al.*^8^ developed a critical scaling relation, based on finite-size scaling effects in percolation theory (see Supplementary Methods) that relates flow to fracture stiffness at a give observation scale through


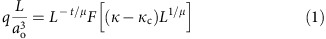


where *q* is the volumetric flow rate (see Supplementary Methods), *L* is the scaled length of the fracture (*L=L*'*/L*_o_ where *L*' is a particular scale and *L*_o_ is the largest scale), *a*_o_ is the mean aperture at the largest scale, *κ* is the fracture-specific stiffness, *κ*_c_ is the critical fracture-specific stiffness, *t/μ* is the flow exponent and *μ* is the geometric correlation exponent (*μ*=4/3). Petrovitch *et al.*^8^ showed that fracture-specific stiffness captures the deformed topology of the fracture under stress, which is the same topology that controls fluid flow through a fracture. This relationship works well for the highly restrictive case of weak spatial correlations in the fracture aperture distribution. However, equation (1) fails to collapse the data (Fig. 3b) for strongly correlated and eroded fractures, which are commonly encountered in the Earth.

To handle strong correlations in fracture aperture distributions, a scaling method must account for fractures with different void volumes, different contact areas and variable spatial correlations in the aperture distribution. In porous media research, interfacial area per volume, *ɛ*, is a common parameter used to capture the distribution of two immiscible fluid phases^25^,^26^,^27^. For fractures, the distribution of contact area and void volume affects both fluid flow and stiffness. Similar to *ɛ* for fluid distributions, the ratio of the volume of voids to the void area is related to the topology of the fracture and is equal to the mean aperture of the fracture, *b*. For example, the 5T and 5TX100 patterns have the same contact area but different void volumes, resulting in mean apertures *b*=5.66 and *b*=7.19 μm, respectively (see Supplementary Table 3). However, the differences in mean aperture are not sufficient to explain the differences in flow rates. From percolation studies, macroscopic conductivity is dominated by the critical neck for a network of strongly inhomogeneous conductances^28^,^29^. For the 5T and 5TX100 patterns, the average aperture of the critical neck is 5.4 and 87 μm, respectively. Therefore, a scaling relationship must account for such differences to extend the scaling of equation (1) to be able to describe these systems.

Random aperture distributions that are fractal have no definable correlation lengths. However, the effective transport length *L*_eff_ depends on the correlation function as


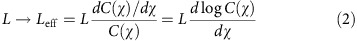


where χ is the length scale, *C(χ)* is the correlation function and *L* is the observation scale (for finite-size scaling). The correlation function of a scale-free pattern has the scale-free property 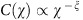 that leads to


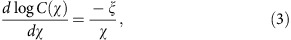


which still contains an indeterminate length scale *χ*. This length is removed by taking the ratio of two effective transport lengths with different correlations, *α*=*ξ*/*ξ*_o_. Therefore, for correlated random flow patterns with scale-free correlation functions, the ratio of the flows scales as the ratio of the correlation exponents.

By including such spatial correlations and accounting for changes in void volume and the critical neck, data collapse for the data in Fig. 3a was achieved using the scaling relationship


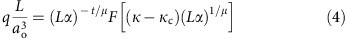


where *α* is the ratio of the correlation exponent at stress *σ* to the correlation exponent at the lowest stress and at the largest scale. The flow *q* is scaled by 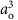, which is redefined as the ratio of the average mean aperture at the largest scale to the average aperture of the critical neck. The threshold parameter *κ*_c_ is the critical stiffness at the critical threshold that is determined by extrapolating the stiffness at the critical area to infinite size (Supplementary Methods). For all fracture conditions, except the highly eroded cases (X25, X50 and X100), *κ*_c_ is independent of scale (see Supplementary Table 4). Even when a fracture is highly channelized, a scale-dependent *κ*_c_ continues to enable data collapse because smaller subsections of the highly channelized fractures require more stress to close the critical path than at larger scales. Changing the moduli of the rock affects the magnitude of the displacement with increasing stress and in turn the magnitude of the specific stiffness. However, different rock moduli, when comparing fractures in different rock types, can be incorporated by multiplying (*κ−κ*_c_) by the ratios of the moduli, that is, the ratio of the Young's moduli *E*_rock1_*/E*_rock2_.

**Figure 4:**
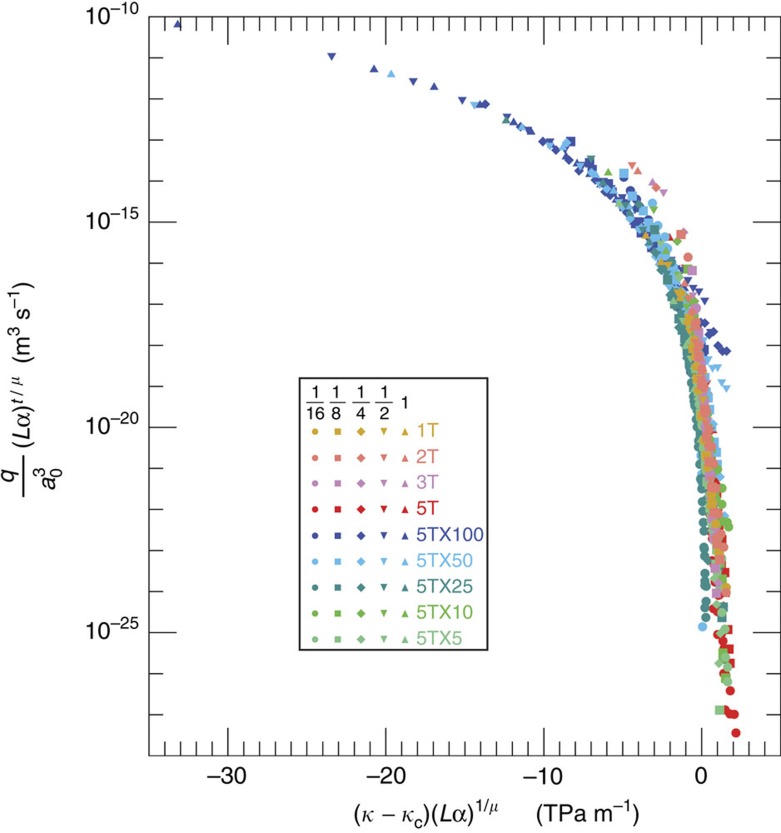
Scaling relationship that produces flow-stiffness data collapse. These data demonstrate the collapse of the data shown in Fig. 3a using equation (4) for the weakly correlated, spatially correlated and eroded fractures, for fracture length scales of 0.0625–1 m. The shape of the symbol indicates the fracture length scale: 0.0625 (circles), 0.125 (squares), 0.25 (diamonds), 0.5 (filled inverted triangle) and 1 m (filled triangle).

Equation (4) was applied to the data shown in Fig. 3a, resulting in the data collapse shown in Fig. 4. The data from all of the simulations for the different correlation lengths and erosions collapse to a single curve. Each curve contains the flow-stiffness data for all five scales for the weakly correlated (1T), spatially correlated (2T, 3T and 5T) and eroded fractures (X5, X10, X25, X50 and X100) (Fig. 4). The collapse exhibits two regimes. For (*κ*−*κ*_c_) (α*L)*^*1/μ*^<−1.0, the scaled flow is in an effective medium regime where the permeability is dominated by the porosity of the fracture. Fractures with highly channelized flow fall into this regime, and the larger-scale fractures at low stress as well. For (*κ*−*κ*_c_) (α*L)*^*1/μ*^≈0.0, the scaled flow is in the critical percolation regime where permeability is controlled by the connectivity of the flow paths. Highly channelized fractures at the largest fracture scale require significant stress to reach the critical percolation regime. Small-scale fractures may contain only one flow path whose connectivity is strongly affected by small changes in stress. The knee in the curve is the transition between the effective medium regime and percolation regime for which both aperture and connectivity play competing or supportive roles in the maintenance of flow. The break in behaviour occurs at (*κ*−*κ*_c_) (α*L)*^*1/μ*^∼−1.0, which captures a fundamental change in the velocity field as it changes from relatively homogeneous flow paths to flow fields at high stress dominated by the critical path^8^.

The collective data collapse of a wide range of fracture topologies and scales provided by equation (4) is an important step forward to capture the complexity of mechanical deformations and their effects on fracture topology that control fluid flow through a fracture. The results presented in this study suggests there is a scaling relationship between fluid flow and fracture-specific stiffness that is valid for a broad range of fracture geometries for the case of laminar flow. To claim universality, all possible thermal, mechanical, geochemical and/or pore-filling fracture alteration scenarios that affect flow and mechanical deformation would need to be explored, as well as flow regimes that span from creeping flow to turbulence. For instance, would this relationship hold for geochemical–geomechanical alterations of fractures that result in hydraulic sealing of fractures? In a recent study, Lang *et al.*^30^ showed through thermo-hydrochemical simulations that for pressure dissolution-dominated compaction, the fracture void space transitions from effective to ineffective porosity as a fracture progressively seals, and that flow ceases long before complete sealing occurs. Similar effects are observed in our numerical simulations of fracture deformation, caused only by mechanical deformation, when flow ceased for contact areas ranging from 40 to 95% depending on the size of the fracture, on the spatial correlations in the aperture distribution, and on the degree of flow channelization. These results suggest that equation (4) has the potential to capture flow-stiffness behaviour even for a fracture undergoing pressure dissolution compaction, but additional work is needed to confirm this.

A key question in both numerical modelling and field monitoring is how to upscale pore or void scale phenomenon to larger scales. For the purpose of modelling subsurface reservoirs, the scaling relationship presented here provides insight into how to appropriately incorporate changing flow rates caused by changing stress. For example, near-surface fractures subjected to less overburden could be treated with an effective medium approach, while fractures at great depth would be in the percolation regime, exhibiting dramatic changes in flow rate with small changes in stress. Furthermore, remote seismic monitoring can probe the subsurface using a range of frequencies enabling it to sample fractured media at difference scales, which could be used with the flow-stiffness scaling relationship developed here, to predict relative flow in subsurface processes.

## Methods

### Fracture generation

A stratified continuum percolation method^16^ was used to generate the void geometry of a fracture (that is, the contact area and variable-aperture voids) without the need to generate two independent rough surfaces. This approach combines the structure of a hierarchical cascade with random continuum percolation (see Supplementary Fig. 1 and Supplementary Table 1) and results in a 2D hierarchical aperture distribution with a tunable spatial correlation. The scale factor used in the cascade controls the spatial correlation length of the aperture distribution.

### Fracture deformation

A combined conjugate-gradient method and fast-multipole method was used to determine fracture deformation. This method is based on the approach of Hopkins^31^ and includes deformation of the asperities, short- and long-range interactions among the asperities and deformation of the solid matrix. The numerical code solves for the local and global deformation of the asperities and the fracture by solving a system of linear equations using the conjugate-gradient method^32^, which reduces the system to matrix-vector multiplication, and the fast multipole method^33^ to increase the speed of the multiplication. This method capitalizes on long-range interactions that are simple to compute with an approximation^21^, whereas short-range interactions require full computation. Deformation was calculated for the five selected scales (1, 1/2, 1/4, 1/8, 1/16 m) as a function of stress (90 stress steps for stresses ranging from 0 to 60 MPa) for each fracture geometry realization and each scale. From the deformation code, the stress-displacement results were used to calculate the fracture-specific stiffness as a function of stress (see Supplementary Fig. 4). For each stress, fluid flow through the deformed fracture was calculated. The output from the deformation code included the deformed fracture aperture array at each stress and were used as input to the flow code and for statistical analysis of changes in the fracture geometry.

### Fluid flow through a fracture

Fluid flow through a fracture as a function of stress was calculated based on the approach of Yang *et al.*^24^, Tran^23^ and Cheng *et al.*^34^ In this model, the aperture distribution is replaced by a network of elliptical pipes that is connected from the inlet to the outlet (Supplementary Fig. 5). Each row of aperture elements perpendicular to the flow is considered in turn and large regions of non-zero apertures are brought together into a single large flow element. Between the rows, flow is calculated based on the analytic solution to flow in an elliptical pipe with conductances based on the apertures^21^,^22^,^23^,^24^,^34^. This approach has order N^2^ linear equations and is preferred over the bilattice grid method, because it is computationally more efficient (run times are four to ten times faster, because the number of elements that define the aperture field is lower). It was also shown to model 2D micro-model experimental data more accurately^34^. The output from the code contains the flow for each fracture realization for all scales at all stresses (for example, see Supplementary Fig. 6).

### Fracture-specific stiffness and critical exponent

The flow and displacement numerical results were binned by stress and then averaged. The average displacement as a function of stress was fit with a curve (see Supplementary Fig. 4) to obtain a smooth derivative as a function of stress and the inverse was used to determine the stiffness at a particular stress. Spanning probabilities were determined as a function of void area fraction and extrapolated to infinite size, to determine the critical area (see Supplementary Fig. 7). After determining the critical area, the value of *q* and *κ*_c_ at each scale for that critical area were calculated. Log–log graphs of *q* at *A*_c_ versus 1*/L* were used to determine the flow exponent, *t/μ*, whereas linear graphs of *κ* versus 1/*L* were used to extract *κ*_c_. Alternatively, *κ*_c_ was extracted from graphs of permeability versus *κ* for the five scales examined in this study (see Supplementary Fig. 7) and the fixed point provides a value of *κ*_c_ (see Supplementary Fig. 8). The correlation exponent was determined from the autocorrelation function for each fracture realization at each scale for each stress. For a given pattern, the average 2D autocorrelation function was used. The results were binned by stress to produce an average value as a function of stress and scale. The mean aperture and critical neck were determined for the largest scale (512 × 512∼1 m) based on the fracture void geometry for the lowest applied stress. The aperture through which the maximum flux occurs was taken as the aperture of the critical neck. The mean aperture was determined by taking the ratio of the void volume to void area. After finding all of the aforementioned parameters, equation (4) was applied to the data, yielding the collapse shown in Fig. 4.

### Code availability

Access to all of the codes used in this study is at the authors' discretion.

## Additional information

**How to cite this article:** Pyrak-Nolte, L. J. & Nolte, D. D. Approaching a universal scaling relationship between fracture stiffness and fluid flow. *Nat. Commun.* 7:10663 doi: 10.1038/ncomms10663 (2016).

## Supplementary Material

Supplementary Information Supplementary Figures 1-8, Supplementary Tables 1-4, Supplementary Methods and Supplementary References
