# Supplementary material for: Approaching a universal scaling relationship between fracture stiffness and fluid flow
Source: Nat Commun. 2016 Feb 12;7:10663. doi: 10.1038/ncomms10663 (PMC4754337; doi:10.1038/ncomms10663)
Supplement: Supplementary Information — Supplementary Figures 1-8, Supplementary Tables 1-4, Supplementary Methods and Supplementary References [file ncomms10663-s1.pdf]

Supplementary Figures

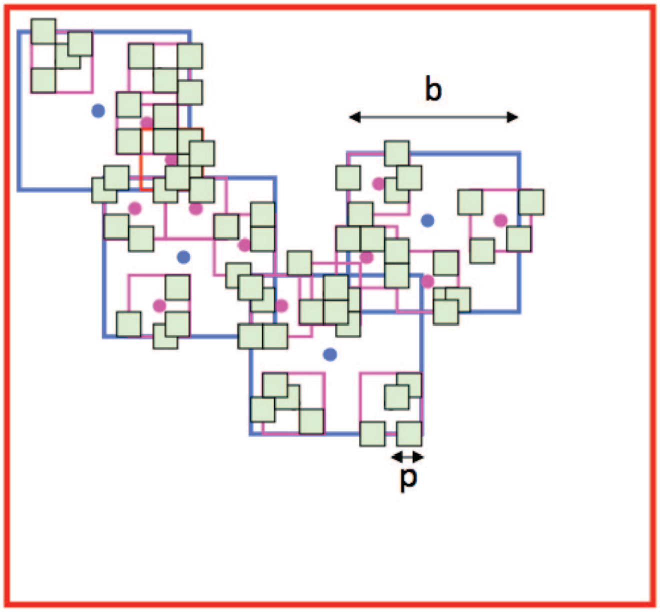

Supplementary Figure 1 Generation of fracture aperture using the stratified percolation approach. This is an example of the generation of a 3 Tier fracture aperture model with $p = 4$ points per tier with a scale factor, $b = 3.78$, between tiers. The first, second and third tiers are shown in red, light blue and magenta, respectively. The light green squares represent the plotted points. Overlaps of the smallest light green squares result in a variable aperture distribution.

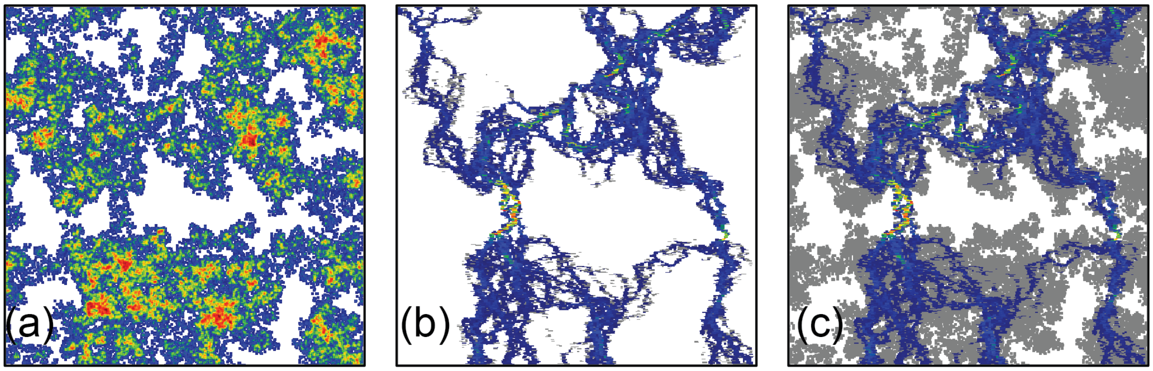

Supplementary Figure 2 Chemical erosion of a fracture aperture distribution. For an initial (a) 5T fracture aperture distribution, (b) the flux distribution is calculated. Chemical erosion is assumed to be transport dominated such that erosion is taken to be proportional to the flux. (c) A composite image of the flux path and initial pattern (shown in gray) illustrates the location of the altered apertures.

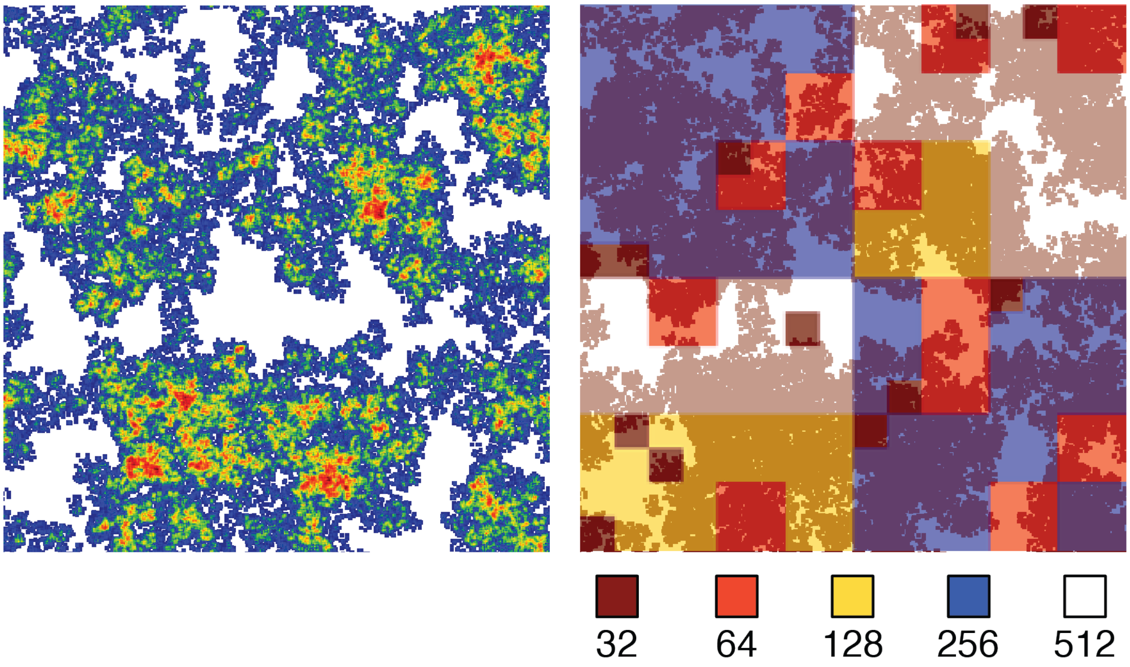

Supplementary Figure 3 Method for taking subsections from a fracture aperture distribution. An example of random sectioning of a 512 x 512 fracture into sections with edge lengths of 32, 64, 128, 256 and 512.

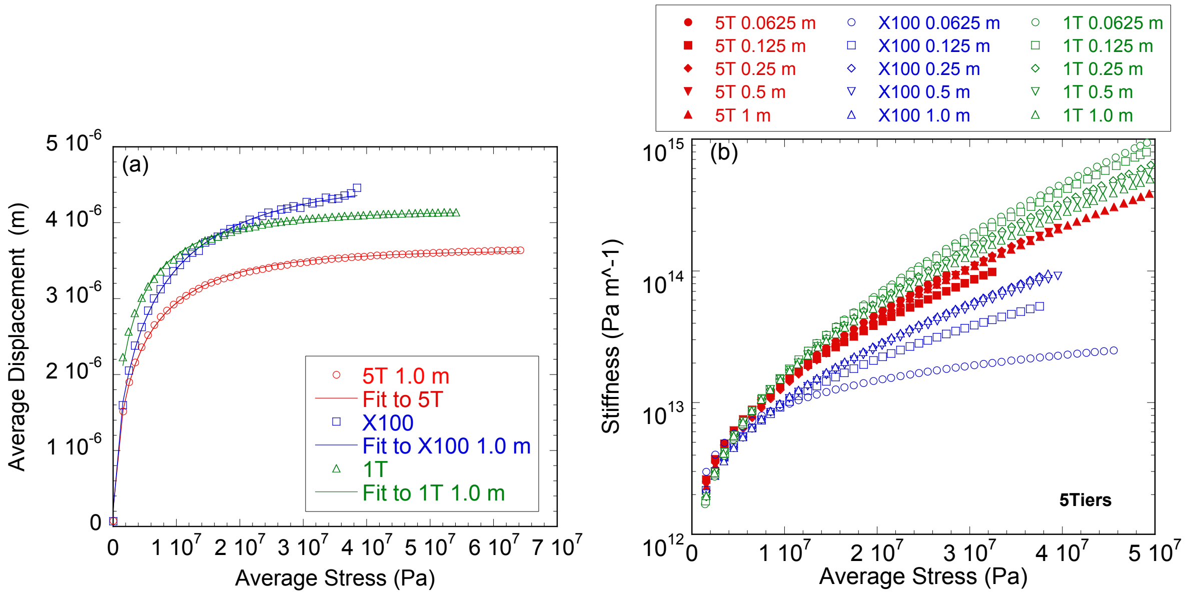

Supplementary Figure 4 Displacement and fracture specific stiffness as a function of stress. (a) Average fracture deformation as a function stress with the fitted curve for fracture realizations from the 1.0 m scale for 5T, X100 and 1T. (b) Fracture specific stiffness as a function of stress for the X100 (open blue symbols), 5T (solid red symbols) and 1T (open green symbols). The shape of the symbol indicates the fracture length scale: 0.0625 (circles ●), 0.125 (squares ■), 0.25 (diamonds ◆), 0.5 (inverted triangles ▼) and 1 m (triangles ▲).

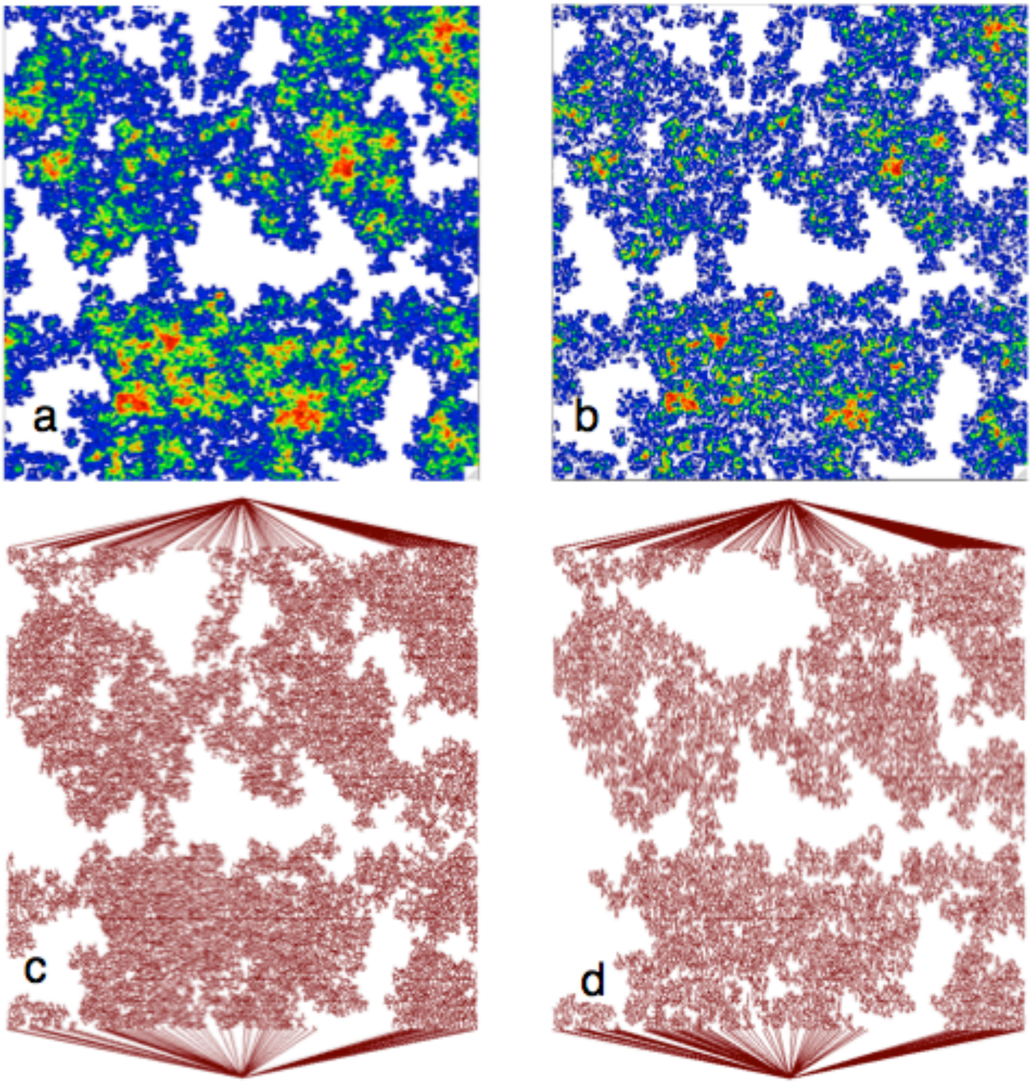

Supplementary Figure 5 Fracture aperture and flow network. Fracture realization and network of elliptical pipes for 5T at low (a &c) and high stress (b &d) used for the flow calculations. The inlet is at the bottom of the network, and the outlet is at the top.

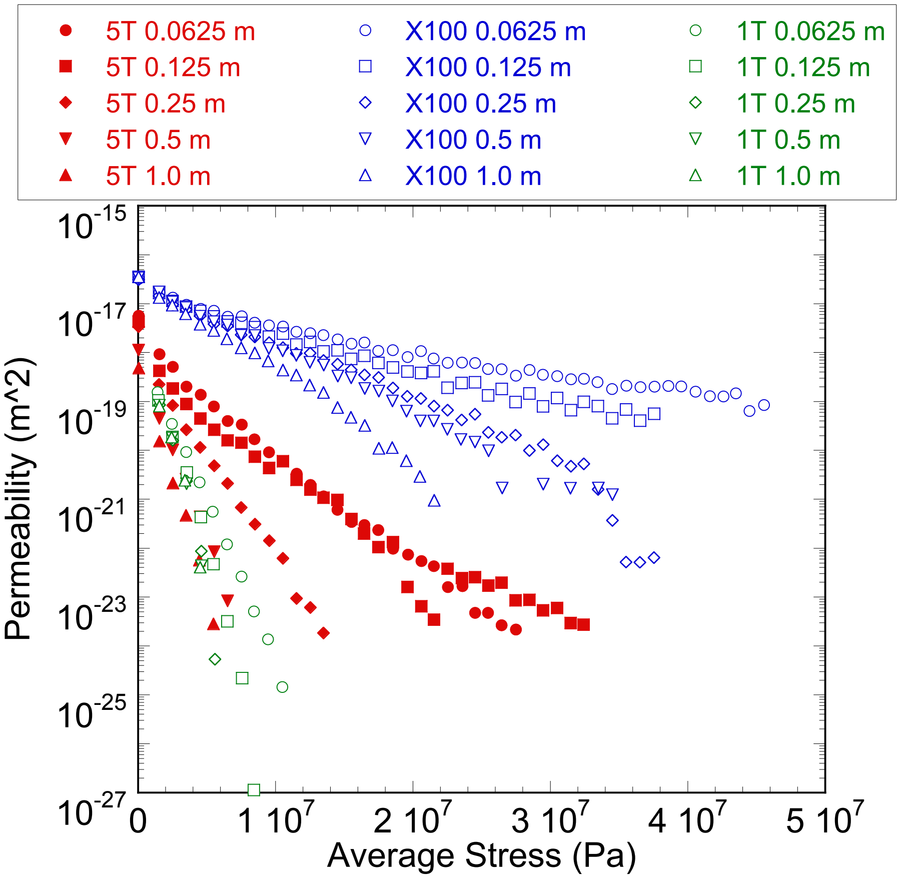

Supplementary Figure 6 Fluid permeability as a function of stress. Fluid permeability is compared as a function stress for the 5T (solid red symbols), X100 (open blue symbols) and 1T (open green symbols) showing the effect of spatial correlations, chemical erosion and fracture scale. The shape of the symbol indicates the fracture length scale: 0.0625 (circles ●), 0.125 (squares ■), 0.25 (diamonds ◆), 0.5 (inverted triangles ▼) and 1 m (triangles ▲).

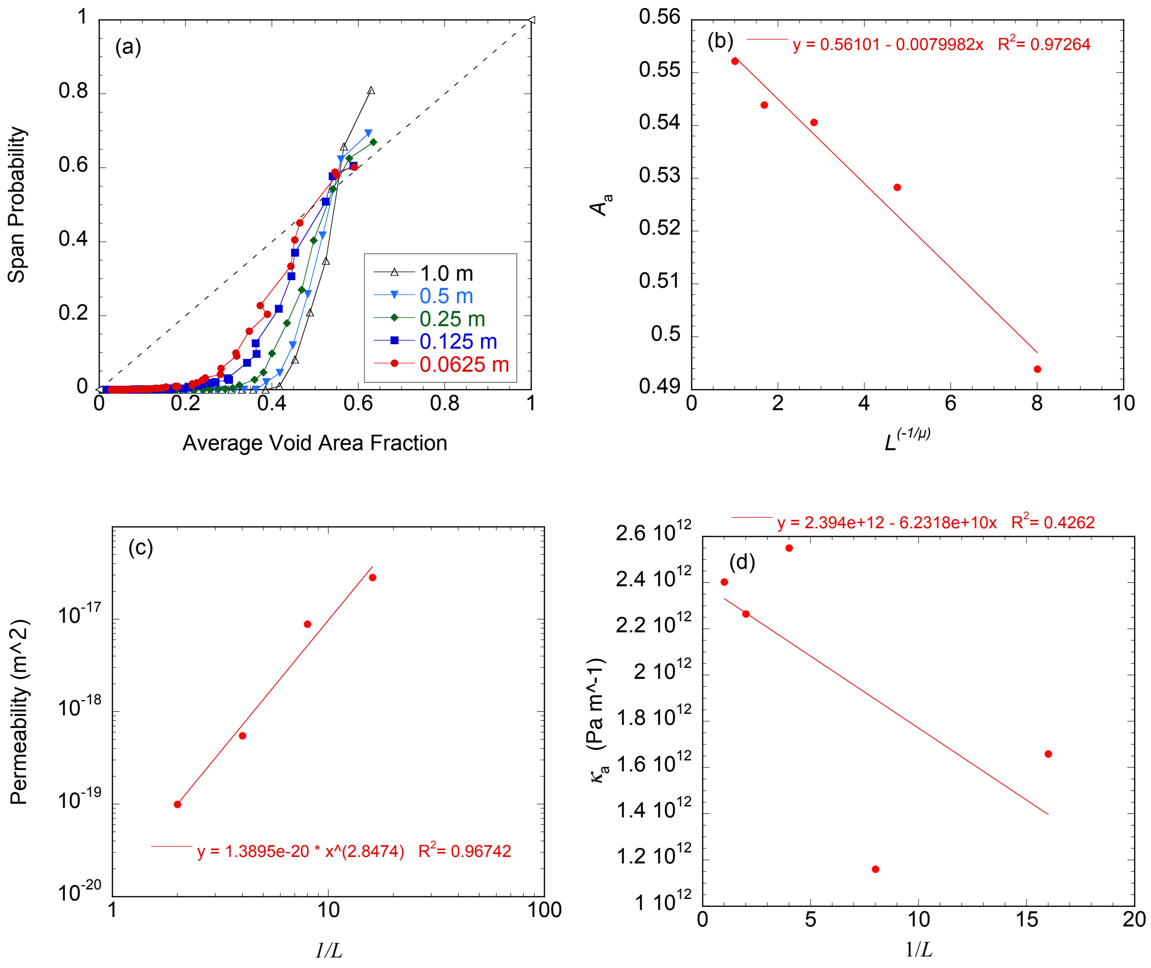

Supplementary Figure 7 Method for determining critical area and critical fracture specific stiffness. For the 5T realizations, (a) spanning probability as a function of average void area fraction for different fracture length scales; (b) area determined from (a) as a function of scaled length, L, raised to the geometric exponent; (c) flow at the critical area fraction as a function of scaled L; and (d) stiffness at the critical area fracture as a function of scaled length.

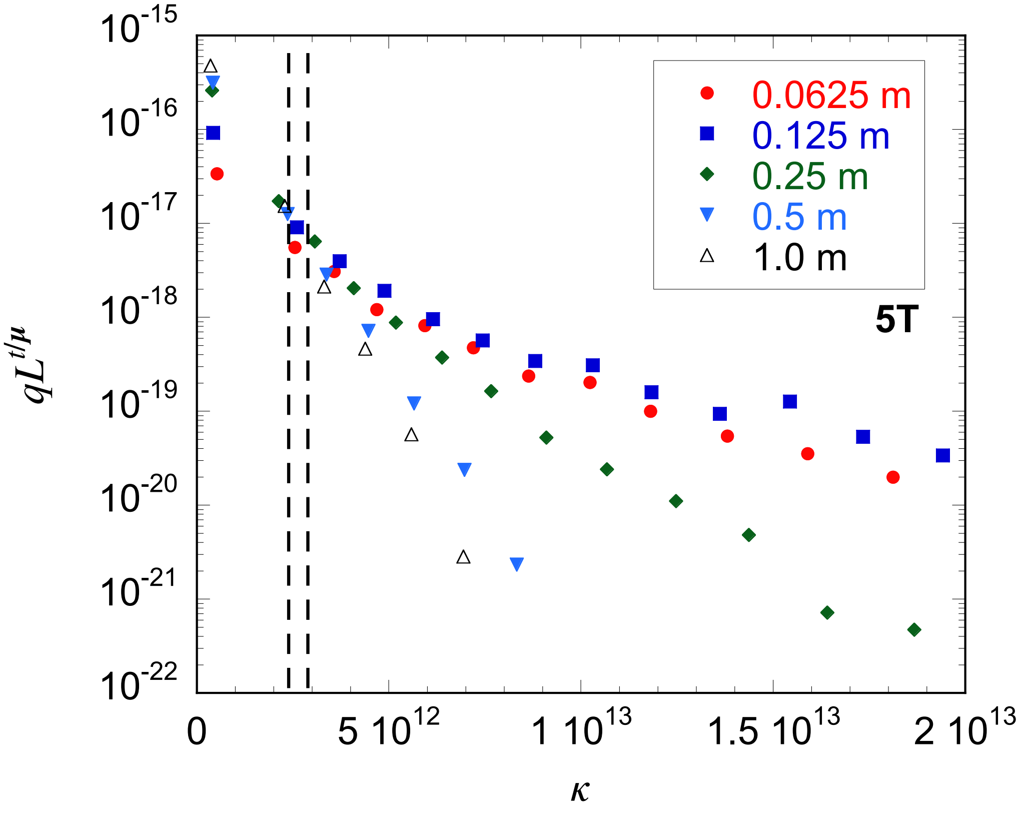

Supplementary Figure 8 Scaled flow versus stiffness for determining κ_c for the 5T fracture as a function of scale. The dashed lines show the region of the invariant fixed point listed in Supplementary Table 3.

Supplementary Tables

Supplementary Table 1 Parameters used to generate the 1T, 2T, 3T and 5T fracture realizations using number of tiers, point size, number of points per tier, and scale factor

Fracture Realization	Number of Tiers	Number of Points	Point Size	Scale Factor
1T	2	260	4	1.0
2T	2	260	4	8.0
3T	3	41	4	4.5
5T	5	9	4	2.64

Supplementary Table 2 Percent of apertures affected by chemical erosion

Fracture Realization	Proportionality Factor	Percent of Apertures Eroded
X5	5	1.99
X10	10	6.32
X25	25	15.45
X50	50	23.13
X100	100	31.87

Supplementary Table 3 Geometric exponent, t/μ flow exponent, critical specific stiffness, κ_c, average mean aperture, $<a_o>$ at the 512 x 512 scale, and average aperture of the critical neck, $<c_o>$, at the 512 x 512 scale

Fracture Realization	μ	t/μ	κ_c* 10^{12} Pa m^-1	κ_c‡ 10^{12} Pa m^-1	$<a_o>$ 10^{-6} m	$<c_o>$ 10^{-6} m
1T	1.333	2.3342	5.5	4.39 - 5.0	4.17	7.40
2T	1.333	2.4677	4.26	4.17	4.40	8.11
3T	1.333	2.7693	3.35	3.56 - 5.06	5.10	8.95
5T	1.333	2.8474	2.39	2.39 - 2.89	5.66	5.37
X5	1.333	3.1386	5.6	4.25 - 5.5	5.69	8.37
X10	1.333	2.6641	6.29	5.75 - 6.25	5.75	12.5
X25	1.333	2.6212	11.2	9.83 - 11.2	5.98	24.5
X50	1.333	2.4884	15.2	14.0	6.37	45.6
X100	1.333	2.7406	38.1	25.0 – 35.3	7.20	87.3

*Fitted from κ vs $1/L$ curve (Supplementary Fig. 7c)
‡Determined from the $qL^{t/\mu}$ versus κ curves (Supplementary Fig. 8)

Supplementary Table 4 κ_c as a function of fracture length for the highly-eroded fracture realizations

Fracture Realization	0.0625 m κ_c x 10^{12} Pa m^-1	0.125 m κ_c x 10^{12} Pa m^-1	0.25 m κ_c x 10^{12} Pa m^-1	0.5 m κ_c x 10^{12} Pa m^-1	1 m κ_c x 10^{12} Pa m^-1
X25	22.8	20.8	16.8	13.8	12.8
X50	30.6	29.6	28.0	24.2	20.0
X100	50.0	50.0	45.0	37.5	33.5

Supplementary Methods

Fracture Geometry Generation using the Stratified Percolation Approach

Fracture aperture distributions were generated on a 512 x 512 (or 1 m x 1m) scale using a stratified percolation approach which combines a hierarchical cascade with random continuum percolation. This approach is illustrated in Supplementary Fig. 1 for the construction of a fracture aperture distribution using a 3 Tier model with 4 points per tier with a point size of *p*. In this approach, a 512 by 512 array is initialized to zero and is the 1st tier (red square in Supplementary Fig. 1). Within this 1st tier, 4 locations are randomly selected that form the center of the 2nd tier (blue squares) which is smaller than the 1st tier by a scale factor, b. Within each of the 2nd tiers, 4 random locations (violet circles in Supplementary Fig. 1) are generated that form the center of the 3rd and final tier (violet squares). In each 3rd tier, 4 points or squares of size p are randomly place. Each time a point is located in the array, each pixel of the point is incremented by 1 unit of aperture. When points overlap, the aperture of the fracture increases. The spatial correlations arise from the hierarchical cascade construction through the selection of b. The random location of the points is based on standard random continuum percolation. For the work shown in this paper, the parameters used to generate each fracture type are listed in Supplementary Table 1. Each unit of overlap was taken to be equivalent to 1.0 μm. Details about the geometric, fractal and percolation properties of fracture apertures generated with this approach can be found in Supplemental reference list articles [1-3].

Method for Eroding the Aperture Distribution

Chemical erosion of a fracture can exhibit a range of behavior depending on the relative time scales associated with advection and chemical reactions [4-6]. We simulated chemical erosion of the fracture by assuming that the amount of erosion is proportional to the flux through the fracture [4], i.e. transport dominated. Supplementary Fig. 2a is an example of the initial uneroded aperture distribution for a 5T realization at the lowest stress. The flow simulation code calculates the flux (Supplementary Fig. 2b) through the aperture distribution as described in the Methods section. The amount of erosion was controlled by the factor of proportionality that affected the percentage of apertures (Fig. 2 in the Main Text and Supplementary Table 2) that were eroded as well as the mean aperture and aperture of the critical neck (Supplementary Table 3). Erosion was only performed on the largest scale (512 x 512 ~ 1 m x 1 m scale) followed by subsectioning.

Method for Subsectioning

The 512 x 512 (1 m) fracture realizations were sectioned into smaller fractures with edge length 256, 128, 64 and 32 (0.5, 0.25, 0.125 and 0.0625 m) to study flow and stiffness as a function of fracture length. For 100 fractures, this would create 65,536 fractures at the 0.0625 m scale. To use the full number of fracture realizations at the 0.0625 m scale would be computationally time prohibitive. Therefore, the number of realizations were scaled with fracture length such that 100, 200, 400, 800 and 1600 realizations were used for 1, 0.5, 0.25, 0.125 and 0.0625 meter scales, respectively (for 512, 256, 128, 64, 32). The subsections were taken randomly from the hundred 512 x 512 realizations. Supplementary Fig. 3 gives an example of the sub-sectioning for a 5T fracture pattern. To section, first the number of subsections, *N*, of size, *S*, in a 512 x 512 pattern,

S_{512}, was determined by $N=(S_{512}/S)^2$. The total number of subsections was multiplied by 100 to yield a total of 100*N* subsections that were assigned a number between 1 to 100*N*. A random number generator was used to select subsection numbers. Care was taken to ensure that any given subsection was not selected more than once.

Fracture Deformation

From theoretical analysis, Cook [7] showed that fracture deformation must include the deformation of the asperities in contact between two fracture surfaces, the deformation of the rock matrix adjacent to the fracture as well as the interaction among asperities. We used the numerical approach of Hopkins[8] to account for the full deformation of the fracture and the matrix. The details and approach of the deformation code are given in articles [9,10] in the Supplemental reference list.

In this section we provide additional details about the process to determine fracture specific stiffness (Supplementary Fig. 4b) as a function of stress from the fracture displacement – stress curve (Supplementary Fig. 4a). Supplementary Fig. 4a contains the average fracture displacement as a function of average stress. The deformation and stress data for this example are the averages from the 100 fracture realizations used at the 1.0 m scale. When taking the averages, the data were binned on stress and then averaged. The data were fitted by a smooth function.

In 1968, Goodman et al.[11] introduced fracture specific stiffness, κ, to describe the behavior of a fracture under normal stress without detailed analysis of the fracture geometry. Intrinsically, κ depends on the contact area between the two fracture surfaces and on the resulting aperture distribution among regions of contact. Several researchers have analytically computed fracture

specific stiffness by representing a fracture as an interface consisting of a periodic array of flat micro-cracks [12-15]. In these analytical approaches, fracture displacement depends on the micro-crack length, spacing and distribution, on the moduli of the rock matrix and on the applied stress. The specific stiffness is taken as the inverse of the derivative of the displacement as a function of applied normal stress, and has units of force/volume.

Supplementary Fig. 4a shows the fracture specific stiffness calculated for the 1 m scale (triangles) and the other scales used in this study. This approach was applied at all scales and for all fracture realizations used in this study. For this analysis, the rock properties of granite were assumed (Young's modulus, E= 60 GPa, Poisson's ratio ν= 0.25). As noted in the Main text, the moduli of the rock affects the magnitude of the specific stiffness. In the flow-stiffness relationship among fractures in different rock types, the quantity (κ - κ_c) should be multiplied by the ratio of the moduli, for example E_{rock1}/E_{rock2}, where E_{rock1} is the reference rock type.

Fluid Flow

Fluid flow through a variable aperture fracture is often simulated using a "local cubic law" approach[16,17]. In that approach, a fracture is discretized into a series of cells representing parallel plates with locally constant apertures and the Reynolds equation, a two-dimensional approximation for flow between parallel plates, is applied to each cell. For Reynolds numbers $<$ 1 and other geometric constraints, this approximation leads to small deviations in the calculated flow rate from the full Navier-Stokes solution[18]. In the study presented here, the aperture distribution is replaced by a network or graph of elliptical pipes based on the approach of

references [9,10,19-22]. This elliptical pipe approach was experimentally validated [19] and found to be more accurate than using a local cubic law approach.

Supplementary Fig. 5 provides examples of the network of elliptical pipes used to calculate fluid flow through the fracture. After fracture deformation, the aperture information is used in the flow code that constructs a network of elliptical pipes from the inlet to the outlet. The density of pipes decreases with increasing stress as regions of the fractures come into contact or are disconnected. Flow through each pipe is given by $q = \Delta P/R$, were ΔP is the head and R is the resistance (inverse of conductivity). The resistance of each elliptical pipe is given by

$$R = \frac{4 f \mu \Delta l \sqrt{K}\left(K+1\right)}{\pi a} \quad (1)$$

where

$$f = \frac{\left(a_1 b_1 + a_2 b_2\right)}{2A_{\text{avg}}} \text{ and } K = \frac{a^2}{h^2}. \quad (2)$$

The parameters a_i and b_i are the major and minor axes of the two ellipses between rows i = 1 and 2. H is half the maximum aperture of the larger ellipse, a is the average minor axis between rows, and A_{avg} is the average area of the two ellipses. A sparse system of linear equations is generated of $O(N^2)$ for an N x N array of apertures. Additional details on the approach can be found in Supplemental reference list article [9]. Examples of the permeability as a function of

stress are shown in Supplementary Fig. 6 for the 5T, X100 and 1T fracture realizations as a function of scale.

Percolation Analysis Approach

Properties of a system become scale dependent when a system is in the critical regime. From percolation theory, the functional form of a physical property χ that scales critically is

$$\chi \propto L^{-\alpha/\mu} F\left[\left(p - p_{\mathrm{c}}\right) L^{1/\mu}\right] \quad (3)$$

where L is the length scale of the system, μ is the correlation exponent, α is the critical exponent associated with the property χ, and p and p_{c} are occupation and critical occupation probability, respectively. F is a universal function that is specific to the process and is valid at all scales. When applying equation 3 to the flow properties of fractures, the occupation probability is replaced with void area fraction, A, leading to

$$q \propto L^{-t/\mu} F\left[\left(A - A_{\mathrm{c}}\right) L^{1/\mu}\right] \quad (4)$$

where t is the transport exponent. Petrovitch et al.[20] demonstrated numerically that fracture specific stiffness is a surrogate for void area fraction because it captures the deformed topology of the fracture, including both the change in contact area and the aperture distribution. The use of κ as a surrogate enabled the formulation of a scaling relationship between flow and fracture specific stiffness (see equation (1) in Main text). In this paper, we extended the functional form of equation (1) in the main text to equation (4) in the main text to account for spatial correlation in void space geometry.

In the following sections, additional information is supplied on the approach to determine the flow exponent (t/μ), the critical stiffness (κ_c), average mean aperture ($<a_o>$) and average aperture of the critical neck ($<c_o>$).

Exponents and Critical Stiffness

The procedure for finding critical exponents and critical stiffnesses begins by determining the critical area. The average spanning probability (Supplementary Fig. 7a) for all fracture length scales is graphed as a function of average void area fraction. Next, a line with unity slope is used to determine the area, A_a, at each scale. From the plot of A_a versus $L^{-1/\mu}$, a linear fit is used to determine the critical area A_c, which, in the example shown in Supplementary Fig. 7b, is $A_c \sim 0.561$. The quantities q and κ_c at the critical area are found for each scale. The flow exponent t/μ is determined from a power law fit to the permeability versus $1/L$ curve (Supplementary Fig. 7c) and for the 5T fractures is equal to 2.8474. The exponents for all fracture realizations are listed in Supplementary Table 3. The critical stiffness was determined from a linear fit to the κ_a (stiffness at the critical area) $1/L$ curve, which gives an intercept of $\kappa_c = 2.394 \times 10^{12}$ Pa m^-1 (Supplementary Fig. 7d). The κ_a (stiffness at the critical area) $1/L$ curve is noisy. Therefore, a second approach for determining κ_c is from the scaled flow ($qL^{t/\mu}$) graphed as a function of fracture specific stiffness for the different fracture length scales. In Supplementary Fig. 8, the scaled flow – stiffness curves exhibit a fixed point where the flow at all scales are equal. The κ at this invariant point is taken as κ_c. Supplementary Table 3 provides a comparison of the κ_c determined using the two approaches.

Fracture realizations 1T, 2T, 3T, 5T, X5, and X10 used the same κ_c at all scales. For the highly eroded fracture realizations (X25, X50 and X100), a constant κ_c was not sufficient to collapse the data. Supplementary Table 4 lists the κ_c as a function of scale for X25, X50 and X100. The lack of a fixed κ_c for channelized fractures is linked to the fact that $(qL^{t/\mu})$ vs κ_c curves do not exhibit an invariant fixed point for all scales.

Mean Aperture, Critical Neck and Correlation Exponent

The extended scaling relation uses average mean aperture, ($<a_o>$) and the average critical neck ($<c_o>$) from the 1 m fracture scale. The average mean aperture was calculated by finding the void volume and void area for each 1m scale fracture realization at the lowest stress. The average of the ratio of void volume to aperture for 100 realizations was taken as ($<a_o>$). The ($<c_o>$) was determined from the flux field for the aperture distribution at the lowest stress. The aperture with the largest flux value was taken as the critical neck.

An autocorrelation analysis was performed on the deformed apertures for all stresses and for all fracture length scales. For a given pattern, the average autocorrelation function was found and fit with the function

$$F = \frac{m_o}{r^{m_1}} + m_2 \qquad (5)$$

where r is distance and m_i are fitted constants. The correlation exponent was taken as

$$\xi = \frac{1}{m_1}. \qquad (6)$$

The extended scaling relation uses an average normalized exponent where $\alpha = \frac{\xi(\sigma)}{\xi_o}$ and ξ_o is the exponent at the 1m scale for the lowest stress.

Supplementary References

1 Nolte, D. D., Pyrak-Nolte, L. J., and Cook, N. G. W. The fractal geometry of flow paths in natural fractures in rock and the approach to percolation. *Pure and Applied Geophysics* **131,** 111-138 (1989).

2 Nolte, D. D., & Pyrak-Nolte, L. J. Coexisting two-phase flow in correlated two-dimensional percolation. *Physical Review E* **56,** 5009-5012 (1997).

3 Nolte, D. D. & Pyrak-Nolte, L. J. Stratified continuum percolation - Scaling geometry of hierarchical cascades. *Physical Review A* **44,** 6320-6333 (1991).

4 Ameli, P., Elkhoury, J. E., Morris, J. P. & Detwiler, R. L. Fracture permeability alteration due to chemical and mechanical processes: a coupled high-resolution model. *Rock Mech. Rock Eng.* **47,** 1563-1573 (2014).

5 Szymczak, P. & Ladd, A. J. C. Microscopic simulations of fracture dissolution. *Geophysical Research Letters* **31**, L23606 (2004).

6 Upadhyay, V. K., Szymczak, P. & Ladd, A. J. C. Initial conditions or emergence; what determines dissolution patterns in rough fractures?. *Journal of Geophysical Research - Solid Earth* **120,** 6102-6121 (2015).

7 Cook, N. G. W. Natural joints in rock: Mechanical, hydraulic, and seismic behavior and properties under normal stress. *International Journal of Rock Mechanics and Mining Sciences* **29,** 198-223 (1992).

8 Hopkins, D. L. *The Effect of Surface Roughness on Joint Stiffness, Aperture and Acoustic Wave Propagation* (University of California, Berkeley, 1990).

9 Petrovitch, C. L. *Universal Scaling of Flow-Stiffness Relationship in Weakly Correalted Fractures* (Purdue University, West Lafayette, Indiana, 2013).

10 Pyrak-Nolte, L. J. & Morris, J. P. Single fractures under normal stress: The relation between fracture specific stiffness and fluid flow. *International Journal of Rock Mechanics and Mining Sciences* **37,** 245-262 (2000).

11 Goodman, R. E., Taylor, R. L. & Brekke, T. L. A model for the mechanics of jointed rock. *Journal of Soil MEchanics & Foundations Division of the American Society of Civil Engineers* **94**, 637-659 (1968).

12 Angel, Y. C. & Achenbach, J. D. Reflection and transmission of elastic waves by a periodic array of cracks. *Journal of Applied Mechanics* **52,** 33-41 (1985).

13 Baik, J. & Thompson, R. B. Ultrasonic scattering from imperfect interfaces: a quasi-static model. *Journal of Nondestructive Evaluation* **4,** 177-196 (1984).

14 Myer, L. R. Fractures as collections of cracks. *International Journal of Rock Mechanics and Mining Sciences* **37,** 231-243 (2000).

15 Nakagawa, S., Nihei, K. T. & Myer, L. R. Shear-induced conversion of seismic waves across single fractures. *International Journal Of Rock Mechanics and Mining Sciences* **37,** 203-218 (2000).

16 Brown, S. R. Fluid flow through rock joints: The effect of surface roughness. *Journal of Geophysical Research* **92,** 1337-1347 (1987).

17 Zimmerman, R. W., Kumar, S. & Bodvarsson, G. S. Lubrication theory analysis of the permeability of rough-walled fractures. *International Journal of Rock Mechanics and Mining Sciences & Geomechanics Abstracts* **28,** 325-331 (1991).

18 Brush, D. J. & Thomson, N. R. Fluid flow in synthetic rough-walled fractures: Navier-Stokes, Stokes and local cubic law simulations. *Water Resources Research* **39,** 1085 (2003).

19 Cheng, J. T., Morris, J. P., Tran, J., Lumsdaine, A., Giordano, N. J., Nolte, D. D., and Pyrak-Nolte, L. J. Single-phase flow in a rock fracture: micro-model experiments and network flow simulation. *International Journal of Rock Mechanics and Mining Sciences* **41,** 687-693 (2004).

20 Petrovitch, C. L., Pyrak-Nolte, L. J. & Nolte, D. D. Combined scaling of fluid flow and seismic stiffness in single fractures. *Rock Mechanics And Rock Engineering* **47,** 1613-1623 (2014).

21 Tran, J. J. *Efficient Simulations of Multiphase Flow in Three-dimensional Fracture Networks* (University of Notre Dame, South Bend, Indiana, 1998).

22 Yang, C., Cook, N. G. W. & Myer, L. R. Network modelling of flow in natural fractures. *Rock Mechanics as a Guide for Efficient Utilization of Natural Resources* 57-64 (A.A. Balkema, Rotterdam, 1989).
